# Functional connectivity between the visual and salience networks and autistic social features at school-age

**DOI:** 10.1186/s11689-025-09613-9

**Published:** 2025-04-28

**Authors:** Jessica B. Girault, Tomoyuki Nishino, Muhamed Talović, Mary Beth Nebel, Margaret Reynolds, Catherine A. Burrows, Jed T. Elison, Chimei M. Lee, Abraham Z. Snyder, Mark D. Shen, Audrey M. Shen, Kelly N. Botteron, Annette M. Estes, Stephen R. Dager, Guido Gerig, Heather C. Hazlett, Natasha Marrus, Robert C. McKinstry, Juhi Pandey, Robert T. Schultz, Tanya St John, Martin A. Styner, Lonnie Zwaigenbaum, Alexandre A. Todorov, Joseph Piven, John R. Pruett, for the IBIS Network, Jessica B. Girault, Catherine A. Burrows, Jed T. Elison, Mark D. Shen, Kelly N. Botteron, Annette M. Estes, Stephen R. Dager, Guido Gerig, Natasha Marrus, Robert C. McKinstry, Juhi Pandey, Robert T. Schultz, Martin A. Styner, Joseph Piven, John R. Pruett, J. Parish-Morris, B. Tunç, W. Guthrie, J.J. Wolff, M.R. Swanson, H.C. Hazlett, R. Grzadzinski, T. St. John, D.W.W. Shaw, J.N. Constantino, A. Rocca, C. Chappell, A.C. Evans, L.C. MacIntyre, S. Torres-Gomez, S. Das, K. Truong, H. Volk, SS Jeste, K.E MacDuffie

**Affiliations:** 1 Carolina Institute for Developmental Disabilities, University of North Carolina at Chapel Hill, Campus Box #3367, Chapel Hill, NC USA; 2 Department of Psychiatry, University of North Carolina at Chapel Hill, Chapel Hill, NC USA; 3 Department of Psychiatry, Washington University School of Medicine in St. Louis, St. Louis, MO USA; 4 Center for Neurodevelopmental and Imaging Research, Kennedy Krieger Institute, Baltimore, MD USA; 5 Department of Ophthalmology and Visual Sciences, Washington University School of Medicine in St. Louis, St. Louis, MO USA; 6 Department of Pediatrics, University of Minnesota, Minneapolis, MN USA; 7 Institute of Child Development, University of Minnesota, Minneapolis, MN USA; 8 Department of Radiology, Washington University in St. Louis, St. Louis, MO USA; 9 Easter Seals, UCP, Raleigh, NC USA; 10 Department of Speech and Hearing Science, University of Washington, Seattle, WA USA; 11 Department of Radiology, University of Washington Medical Center, Seattle, WA USA; 12 Tandon School of Engineering, New York University, New York, NY USA; 13 Center for Autism Research, Children’s Hospital of Philadelphia, University of Pennsylvania Perelman School of Medicine, Philadelphia, PA USA; 14 Department of Computer Science, University of North Carolina at Chapel Hill, Chapel Hill, NC USA; 15 Department of Pediatrics, University of Alberta, Edmonton, AB Canada

**Keywords:** Autism, Functional connectivity, MRI, Brain networks, Social behavior

## Abstract

**Background:**

Autism spectrum disorder (ASD) is highly heritable and phenotypically variable. Neuroimaging markers reflecting variation in behavior will provide insights into circuitry subserving core features. We examined functional correlates of ASD symptomology at school-age, while accounting for associated behavioral and cognitive domains, in a longitudinal sample followed from infancy and enriched for those with a genetic liability for ASD.

**Methods:**

Resting state functional connectivity MRIs (fcMRI) and behavioral data were analyzed from 97 school-age children (8.1–12.0 years, 55 males, 15 ASD) with (*n* = 63) or without (*n* = 34) a family history of ASD. fcMRI enrichment analysis (EA) was used to screen for associations between network-level functional connectivity and six behaviors of interest in a data-driven manner: social affect, restricted and repetitive behavior (RRB), generalized anxiety, inattention, motor coordination, and matrix reasoning.

**Results:**

Functional connectivity between the visual and salience networks was significantly associated with social affect symptoms at school-age after accounting for all other behaviors. Results indicated that stronger connectivity was associated with higher social affect scores. No other behaviors were robustly associated with functional connectivity, though trends were observed between visual-salience connectivity and RRBs.

**Conclusions:**

Connectivity between the visual and salience networks may play an important role in social affect symptom variability among children with ASD and those with genetic liability for ASD. These findings align with and extend earlier reports in this sample of the central role of the visual system during infancy in ASD.

**Supplementary Information:**

The online version contains supplementary material available at 10.1186/s11689-025-09613-9.

## Background

Autism spectrum disorder (ASD) is a highly heritable and clinically heterogeneous developmental disorder affecting 1–2% of the population [1, 2]. Developmental variability in both primary symptom domains and associated features is a key challenge in developing treatment targets and interventions to improve quality of life. Neuroimaging markers that reflect variation in autistic behavior may be useful in informing the underlying neurobiological mechanisms and in designing personalized interventions. In the present study, we aimed to identify functional connectivity profiles that correlate with individual variation in behavioral dimensions relevant to the pathogenesis and treatment of ASD in a heterogeneous sample. We examined associations between functional connectivity (fc) and variation in symptomology, accounting for associated behaviors in a sample of male and female school-age children at high familial likelihood (HL) for ASD by virtue of having an older sibling with ASD, as well as a sample of children at low likelihood (LL) for ASD based on having no familial history.

Reflecting the high heritability of ASD, approximately 20% of HL children receive an ASD diagnosis by age three [3]. An additional 30 to 40% exhibit other developmental concerns during toddlerhood [4] and school-age [5]. Prospective structural neuroimaging studies in HL samples revealed differences in brain development that are apparent in ASD beginning in the first year of life and continuing through toddlerhood [6]. These changes involve multiple systems, spanning sensory to higher-order cognitive areas, and include hyper-expansion of the occipital, temporal, and frontal cortices [7], overgrowth of the amygdala [8], and ultimately global overgrowth of the brain [7, 9]. Studies of fc in HL and ASD infants and toddlers report atypical development of sensory, salience, and default mode networks (DMN) [10–16]. Linking structural and functional findings, we reported that variation in the cortical structure, white matter properties, and fc of brain regions and networks involved in visual processing are traceable to familial indices of genetic liability for ASD [17].

Neuroimaging studies of HL samples at school-age are limited. It remains unclear whether differences in brain development documented during infancy persist, or change, across childhood. A handful of small studies during this period suggest that differences in fc of visual networks and the DMN, consistent with findings in HL samples during infancy, are detectable later in development. Lin et al., found increased intrinsic fc between the mid cingulate and bilateral middle occipital gyrus (areas involved in visual processing) and reduced fc between the midcingulate cortex and right inferior frontal gyrus in both males with ASD and their male HL siblings, suggesting shared functional network architecture in the school-age-to-adolescent period in both males with ASD and those with familial genetic liability for ASD [18]. Another small study (n = 14 per group) in adolescent males reported overall hypoconnectivity in ASD and HL siblings, as well as differences in the architecture of the visual network and DMN (increased and decreased number of hubs, respectively) in ASD compared to controls, with HL siblings exhibiting an intermediate phenotype [19].

Functional connectivity studies in community samples of school-age children with ASD, which likely include a wider range of genetic etiologies than familial HL samples, demonstrated both hypo- and hyperconnectivity in distributed brain networks in ASD. This may reflect age-related differences in fc profiles [20, 21], or subgroups within the datasets related to any number of factors including sex or functioning and/or compensation in other developmental or behavioral domains [22, 23]. In the last decade, there has been a greater focus on linking fc differences to variation in symptomology. These studies, while often conducted across broad age ranges (early childhood to adolescence and adulthood), largely converged on differences in fc involving sensory (including visual), salience, and DMN regions reflecting variation in a variety of ASD-related behaviors [22, 24–28]. For example, Ilioska and colleagues reported between-network hypoconnectivity involving sensory (visual and somatomotor) and attention networks and DMN hyperconnectivity when compared to neurotypical controls, with patterns of connectivity between these networks being correlated with social impairments, restricted and repetitive behaviors (RRB), and sensory sensitivities [28]. Buch and colleagues found that visual-salience fc was related to social affect symptoms among individuals with ASD; individuals with greater social deficits exhibited stronger, positive connectivity between the visual and salience networks [27]. This study also reported that the spatial distribution of these fc patterns reflected regional expression profiles of immune- and serotonergic-related genes [27].

Here, we investigated associations between network-level fc and variation in multiple behavioral domains in HL and LL school-age children (ages 8 to 12 years). We used fc MRI (fcMRI) enrichment analysis (EA), which is designed to overcome the limitations of mass univariate testing in brain-wide analyses through tests at the network-pair level [29]. This approach has been employed to detect associations between dimensional behaviors and network-level fc patterns [29–32]. Here we utilize an expanded version of EA [33] to examine associations between network-level fc and multiple behaviors jointly, including core features of ASD (social affect, restricted and repetitive behavior) and associated behaviors that contribute to the observed heterogeneity in HL and ASD phenotypes and fc profiles [34–36]. Given prior work in this sample during infancy [7, 11, 17, 29, 37], and evidence in children and adults [22, 27, 38, 39], we hypothesized that connectivity involving visual networks would be related to ASD behaviors.

## Methods

### Participants

Resting state fcMRI was obtained in school-age children from the Infant Brain Imaging Study, a longitudinal study of infants at HL and LL for ASD across four data collection sites: University of North Carolina at Chapel Hill, Washington University in St. Louis, University of Washington in Seattle, and the Children’s Hospital of Philadelphia. All study procedures were approved by the respective IRBs. The 97 children included in this sample at school-age (Table 1) were recruited as infants beginning in 2007 (HL: n = 63; LL: n = 34), with 15 children (12 male, 3 female; 11 HL, and 4 from LL group) receiving an ASD diagnosis (Supplemental Methods). Site representation was as follows: Chapel Hill: *n* = 34, St. Louis: *n* = 23, Seattle: *n* = 23, and Philadelphia: *n* = 17. All data for this study were collected prior to the COVID- 19 pandemic with standard imaging and behavioral collection protocols (i.e., no use of face masks). A subset of participants took medication(s) on the day of the MRI (*n* = 12), including 6 participants taking psychotropic medications (Supplemental Methods); sensitivity analyses were conducted controlling for medication use (yes vs. no) and results were unchanged.

**Table 1:** Participant Demographics

	**High Likelihood (HL)** (*n* = 63)	**Low-Likelihood (LL)** (*n* = 34)
Sex (male, female)	36, 27	19, 15
ASD Diagnosis (n, %)	11 (17.5%)	4 (11.8%)
Age (mean, SD)	10.29, 0.94 years	9.65, 1.07 years
Parental education level		
Less than College	2 (3.2%)	0 (0%)
Postsecondary Degree	29 (46.0%)	14 (41.2%)
Graduate Degree	32 (50.8%)	20 (58.8%)
Race, Ethnicity (n, %)		
Asian	0 (0%)	1 (2.9%)
More than one race	7 (11.1%)	2 (5.9%)
White	56 (88.9%)	31 (91.2%)
Hispanic	4 (6.4%)	1 (2.9%)

Participant age, sex, race, ethnicity, parental education level, and socioeconomic status are presented for the 97 subjects with school-age fcMRI and behavioral data included in this study. Parental education level reflects the highest education level attained by either parent. Postsecondary is defined as an Associate’s or Bachelor’s degree

### Functional MRI data collection and processing

fcMRI scans were collected at rest while subjects were instructed to fixate on a crosshair. Behavioral training acclimated the child to the scanner environment to optimize their likelihood of remaining still during the scan [40]. fcMRI data underwent a strict processing protocol [29, 32, 41]. Pre-processing applied slice timing correction, bias field inhomogeneity correction, mode 1000 image intensity normalization, and rigid body correction for both within-run and cross-run head movement. TOPUP was used to estimate and apply subject-level epi distortion correction, as described in Andersson et al. [42] and as implemented in FSL [43]. Data were registered to a standard atlas (711 - 2B version of Talairach space) through a 12-parameter affine transform mapping fcMRI to T2w to T1w to the atlas image in a single transformation. Post-processing followed methods described by Power et al. [44]. Head movement was quantified and censored at a 0.2 mm framewise displacement (FD) equivalent [45]. Data were demeaned and detrended. CSF, white matter, and global signals were used as nuisance regressors, in addition to 6 motion parameters (3 translational displacements along X, Y, and Z axes and 3 rotational displacements of pitch, yaw, and roll) and their derivatives [46]. Data were bandpass filtered at 0.009 Hz < f < 0.08 Hz, and spatial blurring was applied at 6 mm full-width at half maximum. All datasets included in this study passed motion/quality control. Each participant contributed at least 7 min of usable fcMRI data across 11.2 min of acquisition time. All scans were reviewed by a neuroradiologist. See Supplemental Methods for further details on behavioral training and imaging parameters.

A total of 121 participants completed scans. Seven were excluded for exceeding motion thresholds. One participant was excluded for a clinically abnormal MRI. An additional *n* = 16 had incomplete behavioral data and were removed. This resulted in a final sample of 97 participants with usable fMRI and complete behavioral data. Eight subjects (3 LL males, 5 HL females; none diagnosed with ASD) fell asleep or appeared drowsy during the scan. We ran analyses with and without these subjects, as described below, since drowsiness and sleep alter fc correlation patterns [47].

Regions of interest (ROIs) and network assignments were drawn from a recently published functionally defined set of 300 cortical, subcortical, and cerebellar spherical ROIs [48]. After rigorous quality control in our sample, 9 ROIs were removed. The 3 ROIs that were not originally assigned to a functional network [48] were excluded, resulting in a total of 288 ROIs in 13 networks (Supplemental Fig. 1). Network naming follows the “300 ROI Set” labeling convention provided by the authors of [48], which is publicly available at https://greenelab.ucsd.edu/data_software.

### Behavioral measures

We focused on six behavioral variables: calibrated severity scores for (1) social affect (SA) and (2) restricted and repetitive behaviors (RRB) from the second edition of the Autism Diagnostic Observation Schedule (ADOS- 2) [49], measures of (3) generalized anxiety (GAD) from caregiver report on the Multidimensional Anxiety Scale for Children Second Edition [50], (4) inattention from caregiver report (CONP) using the third edition of the Conner’s rating scale for ADHD [51], (5) motor coordination as defined by the upper limb score (LIMB) from the Bruininks-Oseretsky Test of Motor Proficiency, Second Edition [52], and (6) matrix reasoning scores (DAS) from the second edition of the Differential Ability Scales (DAS-II) [53]. Participants exhibited variation in each of these domains (Supplemental Fig. 2). The rationale for the selection of each measure and information on interpretation can be found in Supplemental Methods.

### Statistical analysis

We used enrichment analysis (EA) for resolving patterns of functional network connectivity in relation to behavior. EA is robust to non-normal distributions, as is observed in our behavioral data (Supplemental Fig. 2). First, we employed linear regression to screen for associations between fc for ~ 42 k ROI pairs individually (288 ROIs in 13 networks) and primary ASD symptoms (SA, RRB), adjusting for associated behaviors (GAD, CONP, LIMB, DAS) as well as study site, sex, and age at scan, which were all modeled as fixed effects. The screening statistics were partial *F* tests and two-sided *t*-tests for SA and RRB, after adjustment for other variables. EA then summarizes these region-to-region screening values by network pair to assess overall strength of association with the outcomes of interest, in this case SA and RRB.

We used three different EA statistics, looking for evidence of convergence: over-representation analysis (ORA) [54], max-mean, and gene set enrichment analysis (GSEA). Briefly, ORA tests whether the proportion of screening statistics above a given threshold (a 5% threshold was applied), in a given network pair, is higher than expected by chance alone. The max-mean statistic considers the magnitude and direction of *t*-statistics and is computed as the difference between the sum of the positive and the sum of the absolute values of the negative statistics, divided by the number of ROI-pairs in the network pair [55]. GSEA is one of the most widely used EA methods. It not only tests for an excess of large screening statistics within a network pair, but also whether the statistics in that network pair cluster in the tail end of the distribution over all ROI pairs [56, 57]. Due to the complex correlational nature of the data, we used permutation tests to generate *p*-values [58]. Extensive simulation studies revealed that using a significance level of *p* ~ 0.0005 for individual tests yielded a p ~ 0.05 experiment-wide false positive rate. Statistically significant findings that show evidence of convergence across the EA statistics are interpreted and described. Additional information and limitations regarding these methods are presented in the Supplemental Methods.

## Results

**Fig. 1:**
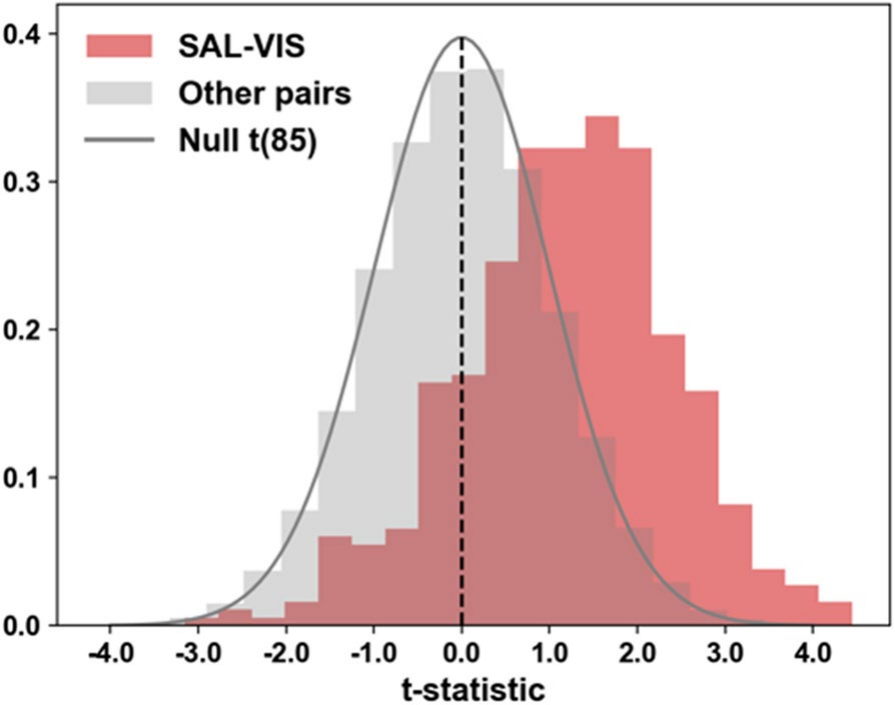
Salience-Visual Functional Connectivity is Associated with Social Affect Scores. ORA results demonstrated a clear shift toward positive values for t-statistics relating fc in SAL-VIS ROI-pairs (red shaded area) to ADOS social affect (SA) scores when compared to the corresponding statistics (grey shaded area) for all other ROI pairs and null t distribution (black line). Results are presented for the entire sample. Analysis based on 250 K permutations of the data

**Fig. 2:**
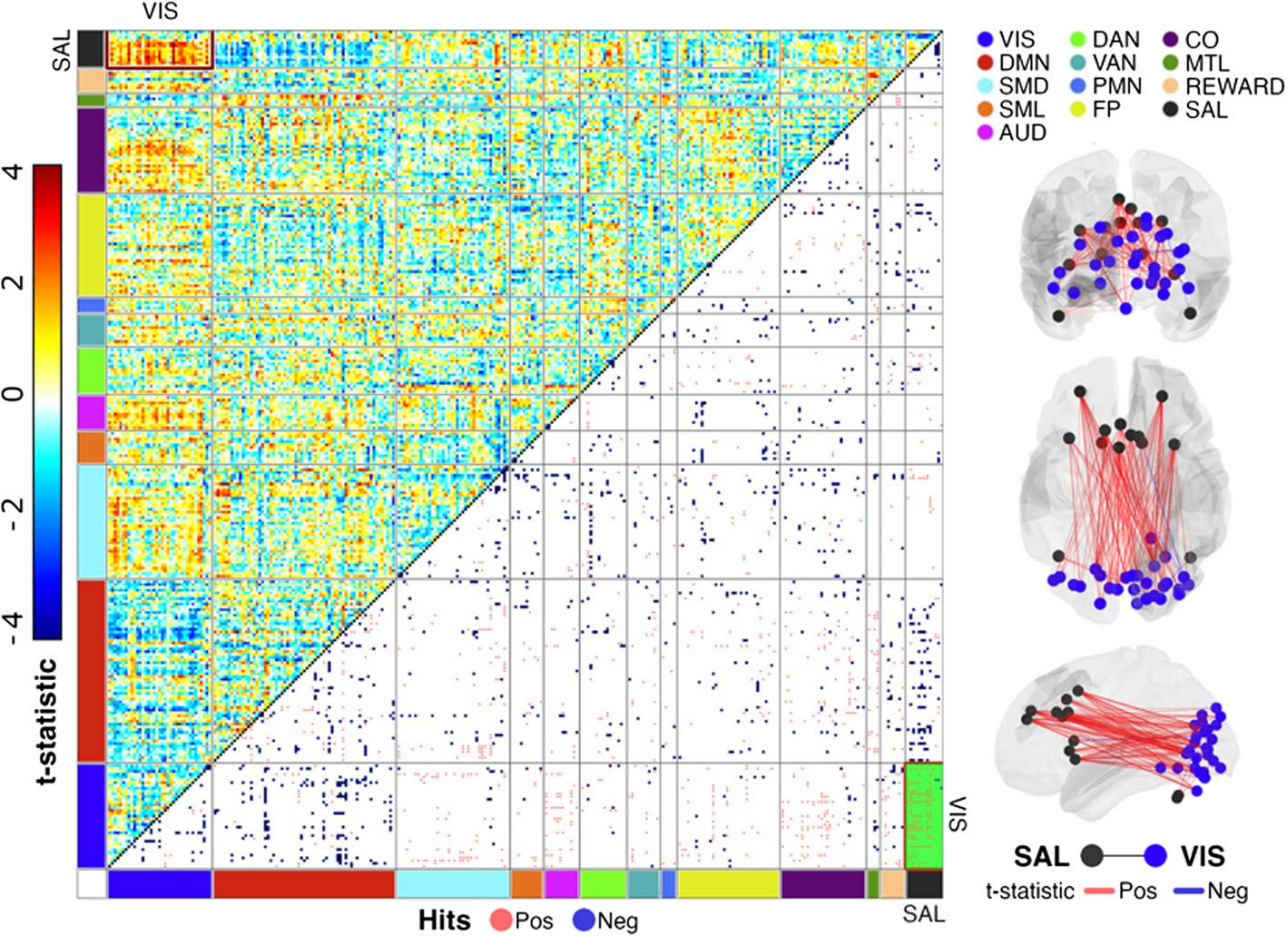
Visualization of Salience-Visual Screening Statistics for Social Affect Scores in Brain Space. Matrix depicts enrichment analysis screening statistics across networks (left panel). Upper triangle displays a heatmap of t-statistics assessing associations between functional connectivity (fc) values and social affect (SA) scores for reach ROI pair (dot in matrix), organized by network: Vis = visual, DMN = default mode network, SMD = somatomotor dorsal, SML = somatomotor lateral, AUD = auditory, DAN = dorsal attention network, VAN = ventral attention network, PMN = parietomedial network, FP = frontoparietal, CO = cingulo-opercular, MTL = medial temporal lobe, REWARD = reward network, SAL = salience. ROIs within each network-network block are organized such that subcortical and cerebellar ROIs are presented first, followed by cortical ROIs. The top left block of the matrix depicts brain-behavior associations in the SAL-VIS network, demonstrating that the vast majority of the positive t-stats (red) are within cortical-cortical ROI pairs. Lower triangles are thresholded to display the strongest brain-behavior associations (top 2.5% of positive and negative t-statistics, or “hits”), colored by whether the t-statistic is positive (red) or negative (blue). The bottom right block of the matrix (green) shows that the vast majority of hits within SAL-VIS are positive, indicating that stronger fc between ROIs in SAL-VIS is associated with higher levels of SA impairment. Visualization of top hits in brain space (right panel)

**Fig. 3:**
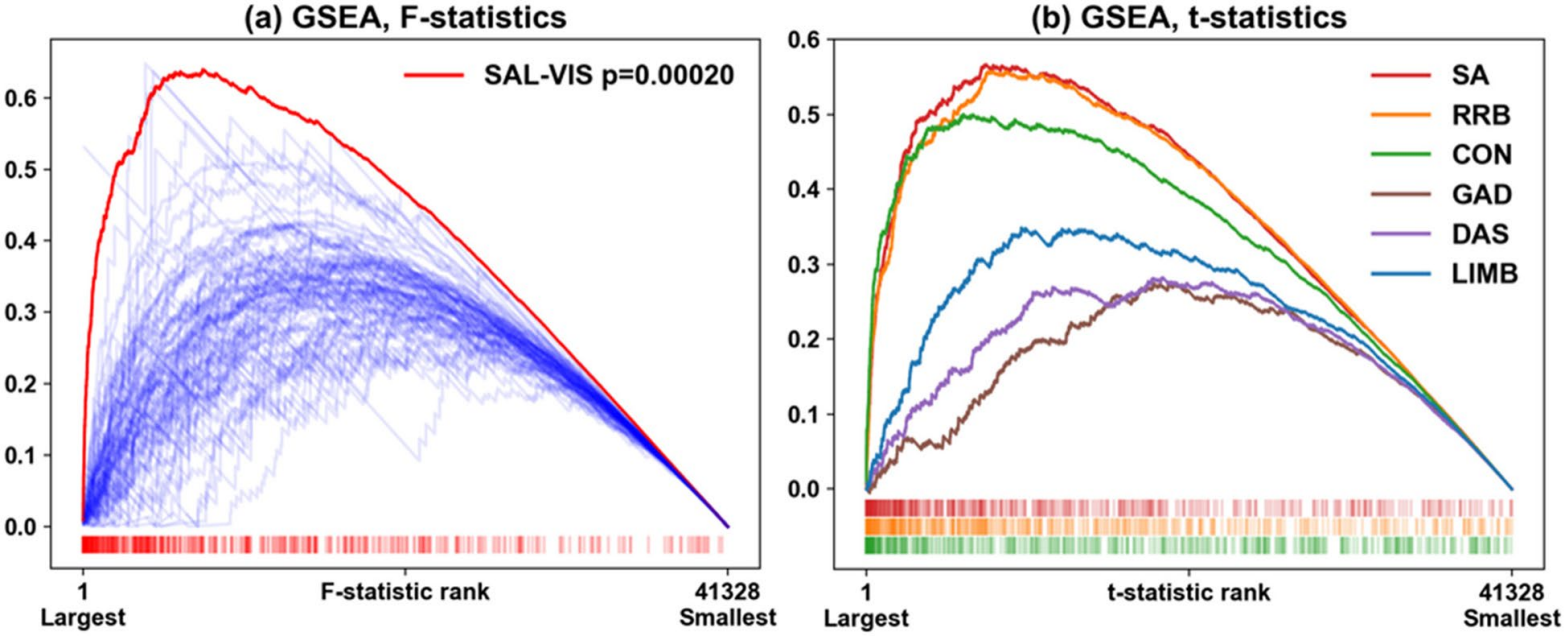
GSEA Profiles Depict Specific Brain-Behavior Associations Unique to the Salience-Visual Network Pair. GSEA profiles (**a**) when screening with partial F statistics for association between fc and SA and/or RRB. There is clustering of large F values in the SAL-VIS network pair (red curve and tick marks); blue curves represent profiles for other network pairs, truncated at 0 for clarity. Smaller network pairs exhibit some instability in the profile. Only the SAL-VIS finding is statistically significant. **b** Profiles when screening SAL-VIS ROI-pairs with t-statistics, one per variable. Clustering of larger t statistics is found for SAL-VIS and SA (*p* = 0.00056), with trend-level findings for RRB (*p* = 0.00072), but not for the other variables (see Table 2). Results presented for the entire sample

All three enrichment statistics implicate the salience (SAL) and visual (VIS) network pair in relation to SA, findings that hold after excluding sleeping/drowsy subjects (Table 2). There were no other significant enrichment signals in any other combinations of behaviors and network pairs, though trend-level associations were observed between SAL-VIS and RRBs (Figs. 1, 2 and 3). Further, including other behaviors of interest improved our ability to detect associations between SAL-VIS and SA (Supplemental Fig. 3).

**Table 2:** Experiment-wide Enrichment Results for Salience-Visual Functional Connectivity

	**All Subjects**			**Excluding n = 8 sleeping subjects**		
	***ORA***	***Max-Mean***	***GSEA***	***ORA***	***Max-Mean***	***GSEA***
**SA**	27.9%	1.30	0.57	26.6%	1.29	0.56
*p-value*	0.00016*	0.00051*	0.00056*	0.00022*	0.00058*	*0.000840*
RRB	25.0%	1.33	0.56	20.0%	1.26	0.53
*p-value*	0.00022*	0.00037*	*0.00072*	*0.0015*	*0.000947*	*0.002520*
SA, RRB jointly	27.2%	N/A	0.65	27.2%	N/A	0.63
*p-value*	0.00001*		0.00020*	0.0001*		0.000360*

Observed values for the ORA (5% threshold), max-mean and GSEA enrichment statistics with p-values in italics: raw permutation p-value are presented, those which meet our *p* ~ 0.0005 threshold for significance are marked with an asterisk (*). Results are given for the entire sample and excluding sleeping subjects (*n* = 8). The *p*-values were determined using independent permutation runs: 25 K permutations for the more compute-intensive GSEA, and 250 K for ORA and max-mean each. Two-sided t-statistics were used to screen for association with SA or RRB individually. Partial F tests were used to screening for association with SA and RRB jointly (d.f. = 2, 85 for the complete sample and d.f. = 2, 77 when excluding sleeping subjects). The expectation for ORA is 5% under the null. Max-Mean is a directional test and cannot be used with F screening statistics. The median max-mean value under the null hypothesis of no enrichment is ~ 0.54 with 0.1% and 99.9% quantile estimates of 0.31 and 1.25 (obtained in separate analyses). The median GSA value under the null is ~ 0.21 (with 0.1% and 99.9% quantiles: 0.17 and 0.60) for the F test and ~ 0.20 (0.18–0.54) for t-tests (separate analysis as well)

SA impairment increased with stronger positive fc for the SAL-VIS ROI-pairs as depicted by the ORA results (Fig. 1). Within SAL-VIS, screening statistics identified 111 ROI pairs (“hits”) associated with SA scores, of which 91% are cortico-cortical, comprised of 47.5% contralateral, 27.7% left- and 24.8% right-lateralized connections (Fig. 2). GSEA also found clustering of SAL-VIS with SA, with *t*-statistics amongst the largest observed across all ROI pairs for any network-pair (Fig. 3a) or behavior (Fig. 3b); no other network-pair was associated with SA (Fig. 3a) and only RRBs were similarly associated with SAL-VIS (Fig. 3b). Analysis with *max-mean* confirms the significant trend towards positive connections for SAL-VIS and SA (Supplemental Fig. 4). Given that at the group level (Supplemental Fig. 1), SAL-VIS fc is weak/minimal, we visually investigated SAL-VIS fc across varying ADOS SA score groups to assess whether SAL-VIS fc may exhibit a unique pattern in those with moderate-to-high SA scores (≥ 5). We observed that SAL-VIS fc is positively shifted for those scoring ≥ 5 (Supplemental Fig. 5). Of note, only 50% of those in our sample scoring ≥ 5 received a diagnosis during the study, indicative of atypical behavioral profiles often observed in HL samples (Supplemental Fig. 2). This suggests that SAL-VIS fc may be uniquely strengthened in those with moderate to high levels of SA impairment, and that this association is not wholly driven by ASD status.

Our EA results also implicated RRBs in a subset of analyses on the full sample (ORA and *max-mean*, but not GSEA), though results did not hold after removing the 8 sleeping subjects (Table 2). The sleeping subjects (none of whom had ASD) had proportions of elevated (≥ 5) and low (= 1) RRB calibrated severity scores comparable to those of the full sample (10% vs 7%, respectively). A joint test for associations between SA and/or RRB and fc yielded significant associations between SA, RRB, and SAL-VIS in the full sample (Fig. 3a) and after excluding sleeping subjects (Table 2), suggesting that RRB may have added value in modeling SAL-VIS fc variation, potentially due to differing associations with fc (e.g., greater RRB impairment, more strongly negative SAL-VIS fc; Supplemental Fig. 6). Further investigation in larger samples will be necessary to confirm RRB links to SAL-VIS fc.

Finally, analyses were conducted to ensure that results were not driven by motion (SAL-VIS includes a large proportion of long-range connections which are particularly susceptible to motion artifacts). We found no evidence that framewise displacement correlated with fc among ROI pairs in SAL-VIS (Supplemental Fig. 7).

## Discussion

We found that functional connectivity between the salience and visual networks was associated with individual variation in social affect symptomology in a school-age sample enriched for ASD and ASD genetic liability. Stronger connectivity between SAL-VIS networks was associated with higher levels of social affect impairment. This aligns with findings during infancy in the same cohort implicating the developing visual system in ASD [7, 8, 11, 17, 37]. We previously reported that infants who develop ASD have differences in the development of cortical structure [7] and white matter properties [59, 60] in regions and fiber tracts involved in visual processing, and that these differences are related to atypical visual orienting during infancy [37], and to familial indices of genetic liability for ASD [17]. We also observed that weakened (less positive) connectivity between VIS and DMN networks in HL infants is related to fewer initiations of joint attention [29] and higher levels of autistic traits in families [17]. These findings align with a wealth of evidence of atypical visual attention and gaze behavior that emerges during infancy in ASD [37, 61]. Further, ASD genes are particularly enriched in the visual cortex [62] and linked to atypical fc patterns in visual areas [38]. Considering this evidence, we hypothesize that differences in the early development of the visual system, related to an increased genetic liability for ASD, initiates a brain-behavior developmental cascade during early infancy that subsequently leads to the emergence of the autistic phenotype [17, 63]. More specifically, our findings linking SAL-VIS to social affect aligns with a growing body of evidence suggesting autistic social behaviors may have their origins in an early developmental cascade involving atypical sensory, and in particular visual, processing [63–65].

Our findings also align with recent studies spanning larger age ranges (late childhood to late adulthood) suggesting that SAL-VIS connectivity may underlie aspects of autistic social impairments across development [22, 27]. Other studies have implicated the salience network in SA and other ASD-related features [22, 24–26], including one which reported machine learning classifiers for predicting ASD outcomes using SAL network connectivity [25]. In particular, Buch and colleagues recently reported that stronger SAL-VIS fc correlated with greater levels of social impairment measured by the ADOS in a multisite sample of ASD and control participants spanning childhood to adulthood [27]. Note that Buch and colleagues used the “Power 2011” ROI set and network labeling scheme [66], while we used an updated ROI set, “Seitzman 2020” [48], where the SAL network is reduced by 9 ROIs (5 of which are assigned to the cingulo-opercular network). Despite this, the results remain highly similar. In both our study and Buch et al., SA scores related to fc between SAL ROIs in the cingulate and frontal cortices and VIS ROIs spanning primary visual and extrastriate occipital cortex. Interestingly, across many fc studies, including this one, SAL-VIS networks show minimal fc at the group level (Supplemental Fig. 1). However, among those in our sample that score in the moderate to high range on SA, SAL-VIS fc was positively shifted (Supplemental Fig. 5), suggesting that this network pair is operating in a potentially unique way in individuals with autistic social impairments that warrants further study.

While the DMN has been repeatedly implicated in brain-behavior studies of related features in this HL sample during infancy [17, 29–32], and in other childhood to adulthood ASD samples [28], we found no link between DMN fc and behavior in this sample at school-age. There are many potential explanations, including developmental shifts in the importance of DMN connections for behavior over time. One notable methodological difference may play a role: infant scans were obtained during natural sleep, while scans at school-age follow-up were obtained while awake, observing a fixation cross. A recent study found stronger positive connectivity between visual cortex and regions of the DMN during eyes closed, and stronger positive connectivity between visual cortex and regions of the SAL network with eyes open [67]. This could partially explain why our infant (sleep) findings often implicated DMN-VIS in relation to joint attention [29], RRBs [31], and familial autistic traits [17], while our school-age (awake) findings implicated SAL-VIS and behavior. It is also possible that our methodological approach (e.g., not analyzing based on diagnosis), or inclusion of a HL sample, may have contributed to differences in results from other work in ASD samples.

We characterized associations between multiple behavioral domains and brain functional connectivity in a data-driven way to reveal robust links between SAL-VIS connectivity and SA impairments in a school-age sample enriched for ASD and subthreshold traits. This approach embraces the heterogeneity observed in HL samples to extend prior work in ASD [27, 28] and demonstrate brain-behavior associations are apparent across diagnostic boundaries. Thus, SAL-VIS connectivity may represent both a neuroimaging marker of risk (stronger connectivity, greater impairment) and potential resilience (weaker connectivity, less impairment) that deserves further study. Further, our approach demonstrated that the inclusion of multiple behaviors simultaneously improved our signal to detect brain-behavior associations, supporting the idea that characterizing both the defining and associated features across a dimension of affectation in both HL and LL subjects can provide more power to detect brain-behavior associations and enhance our understanding of neural phenotypes that likely contribute to an array of neuropsychiatric traits associated with ASD [68]. The ADOS is limited in its ability to characterize meaningful variability in symptoms/behavior in a largely non-ASD sample, as is reflected in the distribution of the SA scores whereby most individuals scored at floor (e.g., exhibited no ASD symptoms). However, there was variation in the moderate to high range among our sample, with half of the individuals with calibrated scores ≥ 5 never receiving a diagnosis of ASD during our study. This reflects the well-documented atypical developmental profiles present in HL children [4, 5] that could be related to ASD features or associated neurodevelopmental disorders (e.g., anxiety, ADHD, intellectual disability)[69]. This suggests that individual differences in SAL-VIS fc may be important for transdiagnostic variation in impairments in social behavior beyond ASD (see also: [70] regarding SAL involvement in depression).

There are limitations related to modeling brain-behavior associations which are high dimensional in nature with a relatively small sample, which call for further investigation of our intriguing but trend-level associations between SAL-VIS fc and RRBs in this sample. While our findings are largely consistent with prior work in their implication of the visual network in familial high likelihood samples [63] and converging pattern of brain-behavior associations linking SAL-VIS fc to social affect [27], there are still inconsistencies in the literature regarding the direction of effect for atypical connectivity patterns in ASD (e.g., hypo vs. hyper connectivity). This difference in findings may reflect the fact that prior studies, both in infants and school-age and adult samples, were largely performed at the group level, and did not involve examining associations with behavior. Because connectivity profiles vary greatly among individuals, reflective of phenotypic variability (see Supplemental Fig. 5), defining connectivity patterns at the group aggregate is likely to obscure important neural signatures that may underlie heterogeneous behavioral features within a given group. This is becoming increasingly evident as the field advances to subject-specific mapping of brain networks [71], where network boundaries have been reported to vary widely across individuals with the same psychiatric diagnosis and relate to clinical symptomology [70]. Relatedly, our study included a relatively short amount of scan time. While this was an intentional design of the study given the population of interest, and in line with typical acquisition protocols in the field, it could impact the reliability of the estimated functional connectivity. Future studies that acquire more fcMRI data per subject [71, 72] could address the question of effects of time on estimates of cross-network connectivity. Another consideration for future work involves understanding the role that medication use may play in modulating brain-behavior associations. A small subset of our participants took medication(s) on the day of the scan, and while we adjusted for this in sensitivity analyses and found no impact on results, future work in larger samples is warranted. Finally, there is a lack of racial and ethnic diversity among participants (who are part of a legacy sample recruited nearly two decades ago), which may limit the generalizability of the findings, though our sample does have unique strengths including a large proportion of HL females (43% of HL sample) and a relatively wide range of cognitive and behavioral ability represented.

## Conclusions

In sum, our findings add to a growing body of evidence implicating visual and salience networks in autistic social behavior that deserves further study. Future work in our sample, and in a new cohort of HL infants for which data collection is underway, will seek to chart the developmental nature of SAL-VIS fc during the period leading up to a diagnosis of ASD to shed light on neurodevelopmental mechanisms that may underlie the emergence of autistic social behaviors. Namely, we are interested in tracking the maturation of the visual system and its connections with other networks, including the salience network, across the first years of life to examine the role that visual system has in shaping attentional behaviors that are fundamental to social learning [63, 65, 73]. Further, future work defining the salience network [74], its boundaries [70], and its functions in ASD samples may be an important avenue for understanding individual variation in autistic social symptoms.

## Supplementary Information

Supplementary Material 1

## Data Availability

The datasets used and/or analyzed in the current study are available from the corresponding author upon reasonable request. The data, in part or in whole, are available via the National Institutes of Mental Health Data Archive (NDA; collection #2775; PI: Piven).

## Abbreviations

ADOS: Autism Diagnostic Observation Schedule
ASD: Autism spectrum disorder
ADHD: Attention deficit hyperactivity disorder
CONP: Inattention scores from caregiver report using the third edition of the Conner’s rating scale for ADHD
DAS: Matrix reasoning scores from the second edition of the Differential Ability Scales
DMN: Default mode network
EA: Enrichment analysis
Fc: Functional connectivity
fcMRI: Functional connectivity MRI
GAD: Generalized anxiety scores from caregiver report on the Multidimensional Anxiety Scale for Children Second Edition
GSEA: Gene set enrichment analysis
HL: High likelihood for autism spectrum disorder
LIMB: Upper limb motor score from the Bruininks-Oseretsky Test of Motor Proficiency, Second Edition
LL: Low likelihood for autism spectrum disorder
MRI: Magnetic resonance imaging/images
ORA: Over-representation analysis
ROI: Region(s) of interest
RRB: Restricted and repetitive behaviors (calibrated severity score from the Autism Diagnostic Observation Schedule)
SA: Social affect (calibrated severity score from the Autism Diagnostic Observation Schedule)
SAL: Salience network
SAL-VIS: Salience-visual network connectivity
VIS: Visual network

## References

[1] Bai D, Yip BHK, Windham GC, Sourander A, Francis R, Yoffe R, et al. Association of Genetic and Environmental Factors With Autism in a 5-Country Cohort. JAMA psychiatry. 2019; 10.1001/jamapsychiatry.2019.1411 PMC6646998 31314057

[2] Maenner MJ, Warren Z, Williams AR, Amoakohene E, Bakian AV, Bilder DA, et al. Prevalence and Characteristics of Autism Spectrum Disorder Among Children Aged 8 Years — Autism and Developmental Disabilities Monitoring Network, 11 Sites, United States, 2020. MMWR Surveill Summ. 2023;72:1–14. 36952288 10.15585/mmwr.ss7202a1 PMC10042614

[3] Ozonoff S, Young GS, Carter A, Messinger D, Yirmiya N, Zwaigenbaum L, et al. Recurrence risk for autism spectrum disorders: a Baby Siblings Research Consortium study. Pediatrics. 2011;128:e488–95. 21844053 10.1542/peds.2010-2825 PMC3164092

[4] Ozonoff S, Young GS, Belding A, Hill M, Hill A, Hutman T, et al. The broader autism phenotype in infancy: when does it emerge? J Am Acad Child Adolesc Psychiatry. 2014;53:398-407.e2. 24655649 10.1016/j.jaac.2013.12.020 PMC3989934

[5] Miller M, Iosif A, Young GS, Hill M, Hanzel EP, Hutman T, et al. School-age outcomes of infants at risk for autism spectrum disorder. Autism Res. 2016;9:632–42. 26451968 10.1002/aur.1572 PMC4826645

[6] Girault JB, Piven J. The Neurodevelopment of Autism from Infancy Through Toddlerhood. Neuroimag Clin N Am. 2020;30:97–114. 10.1016/j.nic.2019.09.009 PMC6878903 31759576

[7] Hazlett HC, Gu H, Munsell BC, Kim SH, Styner M, Wolff JJ, et al. Early brain development in infants at high risk for autism spectrum disorder. Nature. 2017;542:348–51. 28202961 10.1038/nature21369 PMC5336143

[8] Shen MD, Swanson MR, Wolff JJ, Elison JT, Girault JB, Kim SH, et al. Subcortical Brain Development in Autism and Fragile X Syndrome: Evidence for Dynamic, Age- and Disorder-Specific Trajectories in Infancy. Am J Psychiat. 2022;appiajp21090896. 10.1176/appi.ajp.21090896 PMC9762548 35331012

[9] Shen MD, Nordahl CW, Young GS, Wootton-Gorges SL, Lee A, Liston SE, et al. Early brain enlargement and elevated extra-axial fluid in infants who develop autism spectrum disorder. Brain. 2013;136:2825–35. 23838695 10.1093/brain/awt166 PMC3754460

[10] Nair A, Jalal R, Liu J, Tsang T, McDonald NM, Jackson L, et al. Altered Thalamocortical Connectivity in 6-Week-Old Infants at High Familial Risk for Autism Spectrum Disorder. Cereb Cortex. 2021;31:4191–205. 33866373 10.1093/cercor/bhab078 PMC8328203

[11] Liu J, Girault JB, Nishino T, Shen MD, Kim SH, Burrows CA, et al. Atypical functional connectivity between the amygdala and visual, salience regions in infants with genetic liability for autism. Cereb Cortex. 2024;34:30–9. 38696599 10.1093/cercor/bhae092 PMC11065105

[12] Liu J, Okada NJ, Cummings KK, Jung J, Patterson G, Bookheimer SY, et al. Emerging atypicalities in functional connectivity of language-related networks in young infants at high familial risk for ASD. Dev Cogn Neuros-neth. 2020;45: 100814. 10.1016/j.dcn.2020.100814 PMC7341340 32658762

[13] Ciarrusta J, Dimitrova R, Batalle D, O’Muircheartaigh J, Cordero-Grande L, Price A, et al. Emerging functional connectivity differences in newborn infants vulnerable to autism spectrum disorders. Transl Psychiatry. 2020;10:131. 32376820 10.1038/s41398-020-0805-y PMC7203016

[14] Rolison M, Lacadie C, Chawarska K, Spann M, Scheinost D. Atypical Intrinsic Hemispheric Interaction Associated with Autism Spectrum Disorder Is Present within the First Year of Life. Cereb Cortex New York N Y 1991. 2021; 10.1093/cercor/bhab284 PMC8924430 34424949

[15] Tsang T, Green SA, Liu J, Lawrence K, Jeste S, Bookheimer SY, et al. Salience network connectivity is altered in 6-week-old infants at heightened likelihood for developing autism. Commun Biol. 2024;7:485. 38649483 10.1038/s42003-024-06016-9 PMC11035613

[16] Chen B, Linke A, Olson L, Ibarra C, Reynolds S, Müller R, et al. Greater functional connectivity between sensory networks is related to symptom severity in toddlers with autism spectrum disorder. J Child Psychol Psyc. 2021;62:160–70. 10.1111/jcpp.13268 PMC7688487 32452051

[17] Girault JB, Donovan K, Hawks Z, Talovic M, Forsen E, Elison JT, et al. Infant Visual Brain Development and Inherited Genetic Liability in Autism. Am J Psychiat. 2022; 10.1176/appi.ajp.21101002 PMC9356977 35615814

[18] Lin H-Y, Tseng W-YI, Lai M-C, Chang Y-T, Gau SS-F. Shared atypical brain anatomy and intrinsic functional architecture in male youth with autism spectrum disorder and their unaffected brothers. Psychol Med. 2017;47:639–54. 10.1017/S0033291716002695 27825394

[19] Moseley RL, Ypma RJF, Holt RJ, Floris D, Chura LR, Spencer MD, et al. Whole-brain functional hypoconnectivity as an endophenotype of autism in adolescents. NeuroImage: Clinical. 2015;9:140–52. 10.1016/j.nicl.2015.07.015 PMC4556734 26413477

[20] Uddin LQ, Supekar K, Menon V. Reconceptualizing functional brain connectivity in autism from a developmental perspective. Frontiers in human neuroscience. 2013;7. 10.3389/fnhum.2013.00458 PMC3735986 23966925

[21] Burrows CA, Laird AR, Uddin LQ. Functional connectivity of brain regions for self- and other-evaluation in children, adolescents and adults with autism. Dev Sci. 2016;19:564–80. 26750447 10.1111/desc.12400

[22] Keehn RJJ, Pueschel EB, Gao Y, Jahedi A, Alemu K, Carper R, et al. Underconnectivity Between Visual and Salience Networks and Links With Sensory Abnormalities in Autism Spectrum Disorders. J Am Acad Child Adolesc Psychiatry. 2021;60:274–85. 32126259 10.1016/j.jaac.2020.02.007 PMC7483217

[23] Abraham A, Milham MP, Martino AD, Craddock RC, Samaras D, Thirion B, et al. Deriving reproducible biomarkers from multi-site resting-state data: An Autism-based example. Neuroimage. 2017;147:736–45. 27865923 10.1016/j.neuroimage.2016.10.045

[24] Green SA, Hernandez L, Bookheimer SY, Dapretto M. Salience Network Connectivity in Autism Is Related to Brain and Behavioral Markers of Sensory Overresponsivity. J Am Acad Child Adolesc Psychiatry. 2016;55:618-626.e1. 27343889 10.1016/j.jaac.2016.04.013 PMC4924541

[25] Uddin LQ, Supekar K, Lynch CJ, Khouzam A, Phillips J, Feinstein C, et al. Salience Network-Based Classification and Prediction of Symptom Severity in Children With Autism. Jama Psychiat. 2013;70:869–79. 10.1001/jamapsychiatry.2013.104 PMC3951904 23803651

[26] Plitt M, Barnes KA, Wallace GL, Kenworthy L, Martin A. Resting-state functional connectivity predicts longitudinal change in autistic traits and adaptive functioning in autism. Proc Natl Acad Sci USA. 2015;112:E6699–706. 26627261 10.1073/pnas.1510098112 PMC4672806

[27] Buch AM, Vértes PE, Seidlitz J, Kim SH, Grosenick L, Liston C. Molecular and network-level mechanisms explaining individual differences in autism spectrum disorder. Nat Neurosci. 2023;1–14. 10.1038/s41593-023-01259-x PMC11446249 36894656

[28] Ilioska I, Oldehinkel M, Llera A, Chopra S, Looden T, Chauvin R, et al. Connectome-wide Mega-analysis Reveals Robust Patterns of Atypical Functional Connectivity in Autism. Biol Psychiatry. 2023;94:29–39. 36925414 10.1016/j.biopsych.2022.12.018

[29] Eggebrecht AT, Elison JT, Feczko E, Todorov A, Wolff JJ, Kandala S, et al. Joint Attention and Brain Functional Connectivity in Infants and Toddlers. Cerebral cortex (New York, NY : 1991). 2017;27:1709–20. 10.1093/cercor/bhw403 PMC5452276 28062515

[30] Marrus N, Eggebrecht AT, Todorov A, Elison JT, Wolff JJ, Cole L, et al. Walking, Gross Motor Development, and Brain Functional Connectivity in Infants and Toddlers. Cereb Cortex. 2018;28:750–63. 29186388 10.1093/cercor/bhx313 PMC6057546

[31] McKinnon CJ, Eggebrecht AT, Todorov A, Wolff JJ, Elison JT, Adams CM, et al. Restricted and Repetitive Behavior and Brain Functional Connectivity in Infants at Risk for Developing Autism Spectrum Disorder. Biological psychiatry Cognitive neuroscience and neuroimaging. 2019;4:50–61. 30446435 10.1016/j.bpsc.2018.09.008 PMC6557405

[32] Hawks ZW, Todorov A, Marrus N, Nishino T, Talovic M, Nebel MB, et al. A prospective evaluation of infant cerebellar-cerebral functional connectivity in relation to behavioral development in autism. Biological Psychiatry Global Open Sci. 2021; 10.1016/j.bpsgos.2021.12.004 PMC9874081 36712571

[33] Ferguson AS, Nishino T, Girault JB, Hazlett HC, Schultz RT, Marrus N, et al. Statistical properties of functional connectivity MRI enrichment analysis in school-age autism research. Dev Cogn Neurosci. 2025;72: 101534. 40022940 10.1016/j.dcn.2025.101534 PMC11914990

[34] Bartolotti J, Sweeney JA, Mosconi MW. Functional brain abnormalities associated with comorbid anxiety in autism spectrum disorder. Dev Psychopathol. 2020;32:1273–86. 33161905 10.1017/S0954579420000772 PMC8081066

[35] Pua EPK, Malpas CB, Bowden SC, Seal ML. Different brain networks underlying intelligence in autism spectrum disorders. Hum Brain Mapp. 2018;39:3253–62. 29667272 10.1002/hbm.24074 PMC6866443

[36] You X, Norr M, Murphy E, Kuschner ES, Bal E, Gaillard WD, et al. Atypical modulation of distant functional connectivity by cognitive state in children with Autism Spectrum Disorders. Front Hum Neurosci. 2013;7:482. 23986678 10.3389/fnhum.2013.00482 PMC3753572

[37] Elison JT, Paterson SJ, Wolff JJ, Reznick JS, Sasson NJ, Gu H, et al. White matter microstructure and atypical visual orienting in 7-month-olds at risk for autism. Am J Psychiatry. 2013;170:899–908. 23511344 10.1176/appi.ajp.2012.12091150 PMC3863364

[38] Berto S, Treacher AH, Caglayan E, Luo D, Haney JR, Gandal MJ, et al. Association between resting-state functional brain connectivity and gene expression is altered in autism spectrum disorder. Nat Commun. 2022;13:3328. 35680911 10.1038/s41467-022-31053-5 PMC9184501

[39] Hong S-J, de Wael RV, Bethlehem RAI, Lariviere S, Paquola C, Valk SL, et al. Atypical functional connectome hierarchy in autism. Nat Commun. 2019;10:1022. 30833582 10.1038/s41467-019-08944-1 PMC6399265

[40] Nordahl CW, Mello M, Shen AM, Shen MD, Vismara LA, Li D, et al. Methods for acquiring MRI data in children with autism spectrum disorder and intellectual impairment without the use of sedation. J Neurodev Disord. 2016;8:20. 27158271 10.1186/s11689-016-9154-9 PMC4858915

[41] Pruett JR, Kandala S, Hoertel S, Snyder AZ, Elison JT, Nishino T, et al. Accurate age classification of 6 and 12 month-old infants based on resting-state functional connectivity magnetic resonance imaging data. Dev Cogn Neurosci. 2015;12:123–33. 25704288 10.1016/j.dcn.2015.01.003 PMC4385423

[42] Andersson JLR, Skare S, Ashburner J. How to correct susceptibility distortions in spin-echo echo-planar images: application to diffusion tensor imaging. Neuroimage. 2003;20:870–88. 14568458 10.1016/S1053-8119(03)00336-7

[43] Smith SM, Jenkinson M, Woolrich MW, Beckmann CF, Behrens TEJ, Johansen-Berg H, et al. Advances in functional and structural MR image analysis and implementation as FSL. Neuroimage. 2004;23:S208–19. 15501092 10.1016/j.neuroimage.2004.07.051

[44] Power JD, Mitra A, Laumann TO, Snyder AZ, Schlaggar BL, Petersen SE. Methods to detect, characterize, and remove motion artifact in resting state fMRI. Neuroimage. 2014;84:320–41. 23994314 10.1016/j.neuroimage.2013.08.048 PMC3849338

[45] Gratton C, Dworetsky A, Coalson RS, Adeyemo B, Laumann TO, Wig GS, et al. Removal of high frequency contamination from motion estimates in single-band fMRI saves data without biasing functional connectivity. Neuroimage. 2020;217: 116866. 32325210 10.1016/j.neuroimage.2020.116866 PMC7308220

[46] Friston KJ, Williams S, Howard R, Frackowiak RS, Turner R. Movement-related effects in fMRI time-series. Magn Reson Med. 1996;35:346–55. 8699946 10.1002/mrm.1910350312

[47] Laumann TO, Snyder AZ, Mitra A, Gordon EM, Gratton C, Adeyemo B, et al. On the Stability of BOLD fMRI Correlations. Cerebral cortex (New York, NY : 1991). 2017;27:4719–32. 10.1093/cercor/bhw265 PMC6248456 27591147

[48] Seitzman BA, Gratton C, Marek S, Raut RV, Dosenbach NUF, Schlaggar BL, et al. A set of functionally-defined brain regions with improved representation of the subcortex and cerebellum. Neuroimage. 2020;206: 116290. 31634545 10.1016/j.neuroimage.2019.116290 PMC6981071

[49] Lord C, Rutter M, DiLavore P, Risi S, Gotham K, Bishop S. Autism Diagnostic Observation Schedule (ADOS-2). 2nd Edition. Western Psychological Corporation; 2012.

[50] March JS. Multidimensional Anxiety Scale for Children (MASC-2). 2nd Edition. Multi-Health Systems; 2013.

[51] Conners CK, Pitkanen J. Conners Rating Scales (Conners-3). 3rd Edition. Multi-Health Systems; 2008.

[52] Bruininks RH, Bruininks BD. Bruininks-Oserestky Test of Motor Proficiency (BOT-2). 2nd Edition. AGC Publishing; 2005.

[53] Elliott CD. Differential Ability Scales (DAS-II). 2nd Edition. The Psychological Corporation; 2007.

[54] Das S, McClain CJ, Rai SN. Fifteen Years of Gene Set Analysis for High-Throughput Genomic Data: A Review of Statistical Approaches and Future Challenges. Entropy. 2020;22:427. 33286201 10.3390/e22040427 PMC7516904

[55] Efron B, Tibshirani R. On testing the significance of sets of genes. Ann Appl Stat. 2007;1.

[56] Subramanian A, Tamayo P, Mootha VK, Mukherjee S, Ebert BL, Gillette MA, et al. Gene set enrichment analysis: A knowledge-based approach for interpreting genome-wide expression profiles. Proc Natl Acad Sci. 2005;102:15545–50. 16199517 10.1073/pnas.0506580102 PMC1239896

[57] Mootha VK, Lindgren CM, Eriksson K-F, Subramanian A, Sihag S, Lehar J, et al. PGC-1α-responsive genes involved in oxidative phosphorylation are coordinately downregulated in human diabetes. Nat Genet. 2003;34:267–73. 12808457 10.1038/ng1180

[58] Solari A, Finos L, Goeman JJ. Rotation-Based Multiple Testing in the Multivariate Linear Model. Biometrics. 2014;70:954–61. 25269416 10.1111/biom.12238

[59] Wolff JJ, Gu H, Gerig G, Elison JT, Styner M, Gouttard S, et al. Differences in White Matter Fiber Tract Development Present From 6 to 24 Months in Infants With Autism. Am J Psychiatry. 2012;169:589–600. 22362397 10.1176/appi.ajp.2011.11091447 PMC3377782

[60] Wolff JJ, Swanson MR, Elison JT, Gerig G, Pruett JR, Styner MA, et al. Neural circuitry at age 6 months associated with later repetitive behavior and sensory responsiveness in autism. Molecular autism. 2017;8:8. 28316772 10.1186/s13229-017-0126-z PMC5351210

[61] Elsabbagh M, Fernandes J, Webb SJ, Dawson G, Charman T, Johnson MH. Disengagement of Visual Attention in Infancy is Associated with Emerging Autism in Toddlerhood. Biol Psychiat. 2013;74:189–94. 23374640 10.1016/j.biopsych.2012.11.030 PMC3715700

[62] Gandal MJ, Haney JR, Wamsley B, Yap CX, Parhami S, Emani PS, et al. Broad transcriptomic dysregulation occurs across the cerebral cortex in ASD. Nature. 2022;1–8. 10.1038/s41586-022-05377-7 PMC9668748 36323788

[63] Girault JB. The developing visual system: a building block on the path to autism. Dev Cogn Neurosci. 2025;73: 101547. 40096794 10.1016/j.dcn.2025.101547 PMC11964655

[64] Baranek GT, Woynaroski TG, Nowell S, Turner-Brown L, DuBay M, Crais ER, et al. Cascading effects of attention disengagement and sensory seeking on social symptoms in a community sample of infants at-risk for a future diagnosis of autism spectrum disorder. Dev Cogn Neurosci. 2018;29:30–40. 28869201 10.1016/j.dcn.2017.08.006 PMC6414208

[65] Bradshaw J, Schwichtenberg AJ, Iverson JM. Capturing the complexity of autism: Applying a developmental cascades framework. Child Dev Perspect. 2022;16:18–26. 36407945 10.1111/cdep.12439 PMC9673985

[66] Power JD, Cohen AL, Nelson SM, Wig GS, Barnes KA, Church JA, et al. Functional network organization of the human brain. Neuron. 2011;72:665–78. 22099467 10.1016/j.neuron.2011.09.006 PMC3222858

[67] Costumero V, Bueichekú E, Adrián-Ventura J, Ávila C. Opening or closing eyes at rest modulates the functional connectivity of V1 with default and salience networks. Sci Rep. 2020;10:9137. 32499585 10.1038/s41598-020-66100-y PMC7272628

[68] Beauchaine TP, Constantino JN. Redefining the endophenotype concept to accommodate transdiagnostic vulnerabilities and etiological complexity. Biomark Med. 2017;11:769–80. 28891303 10.2217/bmm-2017-0002 PMC5771461

[69] Havdahl KA, Bal VH, Huerta M, Pickles A, Øyen A-S, Stoltenberg C, et al. Multidimensional Influences on Autism Symptom Measures: Implications for Use in Etiological Research. J Am Acad Child Adolesc Psychiatry. 2016;55:1054-1063.e3. 27871640 10.1016/j.jaac.2016.09.490 PMC5131801

[70] Lynch CJ, Elbau IG, Ng T, Ayaz A, Zhu S, Wolk D, et al. Frontostriatal salience network expansion in individuals in depression. Nature. 2024;633:624–33. 39232159 10.1038/s41586-024-07805-2 PMC11410656

[71] Gordon EM, Laumann TO, Gilmore AW, Newbold DJ, Greene DJ, Berg JJ, et al. Precision Functional Mapping of Individual Human Brains. Neuron. 2017;95:791-807.e7. 28757305 10.1016/j.neuron.2017.07.011 PMC5576360

[72] Gordon EM, Chauvin RJ, Van AN, Rajesh A, Nielsen A, Newbold DJ, et al. A somato-cognitive action network alternates with effector regions in motor cortex. Nature. 2023;617:351–9. 37076628 10.1038/s41586-023-05964-2 PMC10172144

[73] Shultz S, Klin A, Jones W. Neonatal Transitions in Social Behavior and Their Implications for Autism. Trends Cogn Sci. 2018;22:452–69. 29609895 10.1016/j.tics.2018.02.012 PMC6554740

[74] Seeley WW. The Salience Network: A Neural System for Perceiving and Responding to Homeostatic Demands. J Neurosci. 2019;39:9878–82. 31676604 10.1523/JNEUROSCI.1138-17.2019 PMC6978945

