# Supplementary material for: Functional connectivity between the visual and salience networks and autistic social features at school-age
Source: J Neurodev Disord. 2025 Apr 28;17:23. doi: 10.1186/s11689-025-09613-9 (PMC12036130; doi:10.1186/s11689-025-09613-9)
Supplement: Supplementary file 1 — Supplementary Material 1 [file 11689_2025_9613_MOESM1_ESM.docx]

**GIRAULT ET AL., SUPPLEMENTAL MATERIAL**

**Table of Contents**

**SUPPLEMENTAL METHODS**

1. Clinical Best Estimate Diagnoses
2. MRI Data Collection and Processing
   1. *Behavioral Training for Scans*
   2. *Medication Use During Scans*
   3. *MRI Data Acquisition*
3. Behavioral Measures
4. Statistical Analyses

**SUPPLEMENTAL FIGURES AND TABLES**

- Supplemental Figure 1: Network Visualization
- Supplemental Figure 2: Distribution of Behavioral Variables
- Supplemental Figure 3: Inclusion of additional behavioral covariates and impact on t-statistics
- Supplemental Figure 4: Max-Mean Results Indicate positive associations for SA, negative associations for RRBs and VIS-SAL functional connectivity
- Supplemental Figure 5: SAL-VIS Connectivity across ADOS SA Score Groups
- Supplemental Figure 6: ORA Results for RRB, associations between RRB and SA findings
- Supplemental Figure 7: Framewise displacement and fc in SAL-VIS ROI pairs

**____________________________________________________________________________________**

**SUPPLEMENTAL METHODS**

**Clinical Best Estimate Diagnoses**

Subjects in this study received a clinical best estimate (CBE) diagnosis at their school-age visit using DSM-5 criteria; they also received a CBE diagnosis at their 2-year-old visit using DSM-IV-TR criteria which were standard at the time. Clinicians incorporated all information gathered throughout the visit to determine whether the child met criteria for ASD. Clinicians provided rationale when a child had high ADOS scores but did not receive a diagnosis. A total of 15 children in the study received a diagnosis of ASD at the 2-year and/or school-age follow-up visit. Of these 15 children, 4 received an ASD diagnosis at both visits (26.7%), 5 gained a diagnosis at school-age (33.3%), and 5 no longer met criteria at school-age (33.3%), and 1 subject received their first diagnostic outcome at school-age (i.e., no prior data available; 6.7%). Two subjects were missing 2-year diagnostic outcomes but were negative for ASD at school-age. See **Supplemental Figure 2** for information on the distribution of ADOS calibrated severity scores.

**MRI Data Collection and Processing**

***Behavioral Training***

Study personnel were trained in behavioral methods by a licensed board-certified behavior analyst (author AMS) based on their previously published methods [1] . A Pre-Visit Structured Interview was conducted with the parents about the participant’s strengths and challenges in lying still in an MRI scanner. A Reinforcement Survey was completed to determine what child-specific reinforcers and rewards would motivate the participant to cooperate in the scanning environment. Parents were mailed home a visual storyboard depicting the steps of the MRI scan and a practice kit (CD of the scanner sounds and earplugs). At the visit, the staff, parent, and participant collaborated on a process of shaping and desensitization in the MRI environment. A mock MRI scanner equipped with a motion sensor was utilized to teach the participant the contingency of remaining still while lying in the enclosed environment inside the scanner bore. The participant was fitted with earplugs, headphones, and chose the movie of their choice while the MRI scan sounds played in the room. When the threshold of the motion sensor was reached, the movie paused and the screen blacked out. The threshold was set to the highest level at first, until the participant understood that they needed to remain still to watch their movie. The threshold was systematically lowered as the mock scan session continued until the participant was watching their movie without interruption while moving less than 1mm. At this point, the participant was deemed ready to move to the actual MRI scanner. During the MRI session, the participant watched their movie during the structural scans. Before the BOLD scans, the video and audio were turned off, and the participant was instructed to fixate on a crosshair on the screen.

***Medication Use***

Of the 97 children included in our datasets (usable fcMRI + complete behavioral data), 12 (12.4%) were reported to have taken medications on the day of the MRI. The medications taken by these 12 subjects are presented below the table below (note, some subjects were taking more than one medication). Psychotropic medications were taken by n = 6 participants (3 HL, 3 LL; 3 Male, 3 Female; 2 ASD) and included selective serotonin/serotonin-norepinephrine reuptake inhibitors (SSRIs, SNRIs), psychostimulants, anxiolytics, and antipsychotic medications. None of the 8 subjects reported to have fallen asleep or been drowsy during the scan took medications.

| **Drug classification** | **Number of participants taking medications in specific category** | **Specific medications** |
| --- | --- | --- |
| Antipsychotic | 2 | Risperidone |
| Antiepileptic | 1 | Lamotrigine* |
| Stimulant | 3 | Dextroamphetamine-Amphetamine; Dexmethylphenidate (2) |
| SSRI | 1 | Sertraline |
| SNRI | 1 | Atomoxetine |
| Anxiolytic | 1 | Buspirone |
| Vitamins | 4 | Fish oil magnesium; Nordic berries; multivitamin; TUMS (Calcium carbonate); “Luna plus”-a sleep aid including melatonin, valerian root, chamomile, lemon balm “and more” |
| Inhaled steroids | 1 | Fluticasone Propionate |
| Antihistamine | 1 | Cetirizine |

*Note: Lamotrigine is primarily used as an antiepileptic, but the one participant taking this drug has no history/current report of seizures; drug assumed to be used for off-label treatment of psychiatric symptoms.

### ***MRI Data Acquisition***

School-age participants underwent a scanning protocol involving structural, diffusion and functional connectivity MRI scans (fcMRI); the structural and fcMRI scan data were used in this study. MRI scans were collected at four sites using the same Siemens (Erlangen Germany) 3T Prisma and Prisma Fit scanners with a 32-channel head coil. Due to potential concerns regarding this population’s ability to remain still for extended periods of time, scan duration was purposefully shortened at the cost of signal-to-noise (SNR). Two short-duration T1-weighted images (MP-RAGE; duration: 3:29; 1 x 1 x 1 mm voxels; TE = 2.03 ms; TI = 1000 ms; TR = 2500 ms; flip angle: 8°; acceleration factor: 4) were averaged to increase signal-to-noise while minimizing scan time (Holmes et al. 1998). A short T2-weighted (SPACE; duration: 2:00; 1 x 1 x 1 mm voxels; TE = 564 ms; TR = 3200 ms; acceleration factor: 4) was also acquired. Four runs of BOLD (duration: 4:09 each; Multiband 6; 2.4 x 2.4 x 2.4 mm voxels; TE = 30 ms; TR = 800 ms) were acquired during eyes-open fixation to perform resting-state functional connectivity MRI (fcMRI) analyses. Cross-site functional connectivity differences were not observed, though site was still included as a nuisance variable.

**Behavioral Measures**

| **Assessment** | **Measure** | **Interpretation** | **Rationale for Inclusion** |
| --- | --- | --- | --- |
| MASC-2 (Caregiver Report) | Generalized anxiety T score | Higher score = greater levels of impairment/ symptoms | Anxiety problems are common in ASD and HL siblings; whether ASD and anxiety traits share overlapping neural signatures is unclear and of scientific interest. |
| Conner’s 3 (Caregiver Report) | Inattention T score | Higher score = greater levels of impairment/ symptoms | Attention problems are common in ASD and HL siblings; whether ASD and attention traits share overlapping neural signatures is unclear and of scientific interest. |
| BOT-2 | Upper Limb Coordination Summed total score | Higher score = better performance | Motor coordination problems are common in ASD and HL siblings. Upper limb coordination measures ball skills requiring the rapid integration of visual information to guide movement, most similar to motor measures often showing differences in ASD. |
| DAS-II | Matrices T score | Higher score = better performance | Measure of non-verbal reasoning involving the perception and application of relationships among abstract figures. Has been shown to differ in ASD. |
| ADOS -2 | Social Affect CSS, RRB CSS | Higher score = greater levels of impairment/ symptoms | Gold standard for ASD symptom measurement in the context of diagnostic evaluation. Available on all subjects in this dataset. Identical to measures used in other recent reports in older samples. NOTE: does have limited range/validity in measuring subthreshold ASD symptoms. |

**Statistical Analyses**

Our statistical approach utilized Enrichment Analysis (EA) to evaluate brain-behavior associations after jointly modeling fc as a function of behavioral features plus covariates (sex, age, study site). We studied three EA statistics, as described in the main text: ORA, max-mean, and GSEA. Here we include a few additional methodological details and limitations of this approach.

First, the distribution of ORA statistics is intractable, due to the complex correlational structure of the data. We thus used permutation tests, as described in Solari and colleagues [2]. Note that these authors used rotation rather than permutation tests. Preliminary work showed that the two approaches, for this application, yielded essentially identical results.

Our analytic approach was well suited for the task at hand but has limitations that should be noted. First, there are a number of other EA statistics which we did not consider. We chose three methods that are quite different in their approach to EA, which yielded converging findings. Second, the use of a linear model is a limitation. We are limited there by the sample size and the skewed distribution of the variables of interest. Modeling, e.g., SA and RRB as a function of connectivity (rather than the reverse) may have been preferable, but not feasible with our limited sample size. The variables themselves show significant correlations. We verified that multicollinearity was not an issue based on the following: (1) correlations between GAD, CON, RRB and SA are significant, but not extreme (*r* = 0.31 to 0.45); (2) variance inflation factors (VIF) were acceptable (BOT2: 1.19; DAS: 1.03; GAD: 1.15; CONP 1.39; RRB: 1.38; SA: 1.41); (3) the condition number of the X’X matrix was 4.4, well below the usual threshold of 20 for concern; and (4) we ran simulation studies to verify that the test statistics under our permutation test procedures (under the null) closely followed that expected from first principles.

Additional limitations include the risk of a false-positive. There, we use a rather strict significance threshold, which we determined yielded an experiment wide ~0.05 risk of a false positive. We conducted extensive preliminary analyses and used a large number of permutations to arrive at accurate estimates of *p-*values and false-positive risks. Finally, if the screening statistics clearly show trends toward positivity for SA and negativity for RRB, none would have survived a multiple-testing correction in pure mass-univariate screening. The signal is at the network-pair aggregate rather than at the individual ROI-pair level.

**SUPPLEMENTAL FIGURES AND TABLES**

**Supplemental Figure 1: Network Visualization**


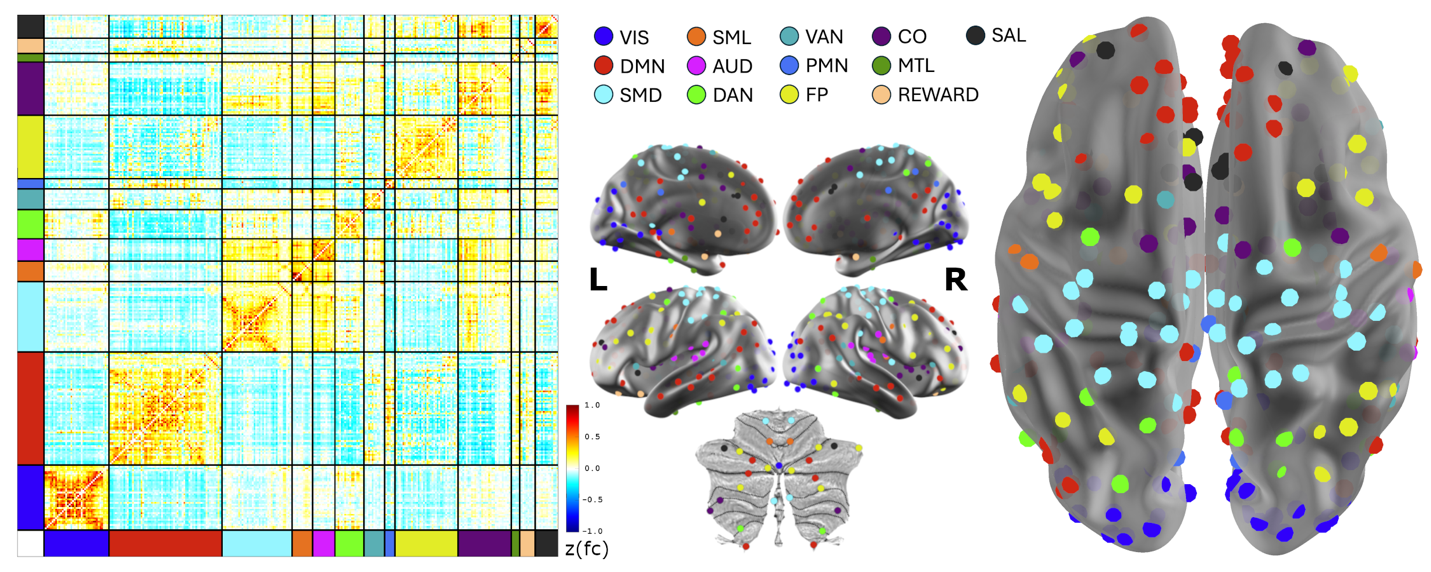


**(Left)** Matrix depicts sample-mean functional connectivity (fc) correlation structure among 288 regions of interest (ROIs) assigned to 13 networks [3]: Vis = visual, DMN = default mode network, SMD = somatomotor dorsal, SML = somatomotor lateral, AUD = auditory, DAN = dorsal attention network, VAN = ventral attention network, PMN = parietomedial network, FP = frontoparietal, CO = cingulo-opercular, MTL = medial temporal lobe, REWARD = reward network, SAL = salience. Color gradient illustrates the strength of fc correlation between ROIs. **(Center/Right)** Functional networks and spherical ROI constituents (colored by network label) are depicted on dorsal, lateral, and medial surfaces. Cerebellar ROIs are depicted on a flattened surface [4].

**Supplemental Figure 2: Distribution of Behavioral Variables**

Distributions of ADOS Social Affect (SA) **(A)** and Restricted and Repetitive Behaviors (RRB) **(B)** are shown for the entire sample and split by group (HL vs. LL and by ASD vs non-ASD (“Neg”)). Of those scoring ≥ 5 on the SA calibrated severity score (n = 16), 50% received an ASD diagnosis at their 2-year and/or school-age visit. Of those scoring ≥ 5 on the RRB calibrated severity score (n = 39), 34% received an ASD diagnosis at their 2-year and/or school-age visit. **C-F** show distributions for the other four behavioral variables of interest for the full sample.

**Supplemental Figure 3: Inclusion of additional behavioral covariates and impact on t-statistics**

**
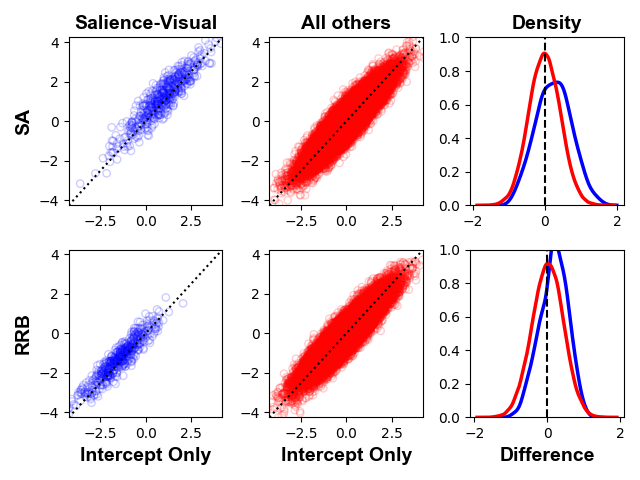
**

**Left Panel:** The enrichment signal for SAL-VIS and SA (top row) and RRB (bottom row) is enhanced when a ‘full model’ is used (SA and RRB, plus other behavioral variables; blue) compared to ‘intercept’ models with only intercept and SA (top left) or RRB (bottom left). **Middle Panel:** This shift is not seen for ROI pairs outside of SAL-VIS (middle panels; red). Visually, the signal increase is seen as a shift above the dashed black line in the scatterplot of t-statistics for each ROI-pair for the full and intercept models (**left/right panels**). **Right Panel:** Signal increases of adding covariates is depicted as a shifts in the density plots of the difference in the t-statistics for full and intercept models. The strengthened t-statistics for the full model is clearest for SA (top), and is less pronounced for RRBs (bottom).

**Supplemental Figure 4: Max-Mean Results Indicate positive associations for SA, negative associations for RRBs and VIS-SAL functional connectivity**


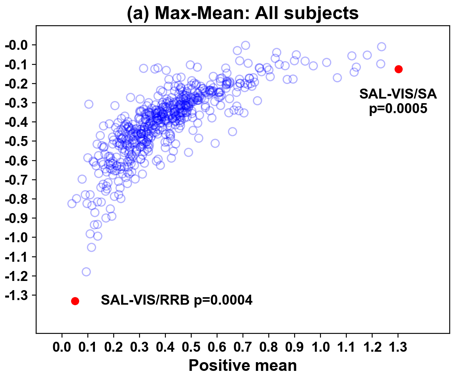
 *
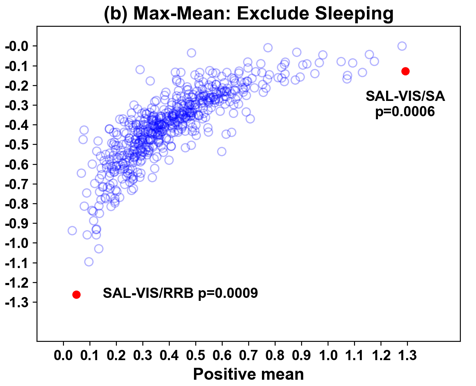
*

Enrichment analysis with *max-mean*: **(a)** all subjects; **(b)** excluding sleeping subjects. For each combination of behavior (DAS, LIMB, CONP, GAD, SA, RRB) and network pair, weighted average of the positive (*x-axis*) and negative (*y-axis*) screening *t* statistics. Only SAL-VIS and SA or RRB meet the experiment-wide significance level in either condition (a) or (b).

**Supplemental Figure 5: SAL-VIS Connectivity across ADOS SA Score Groups**


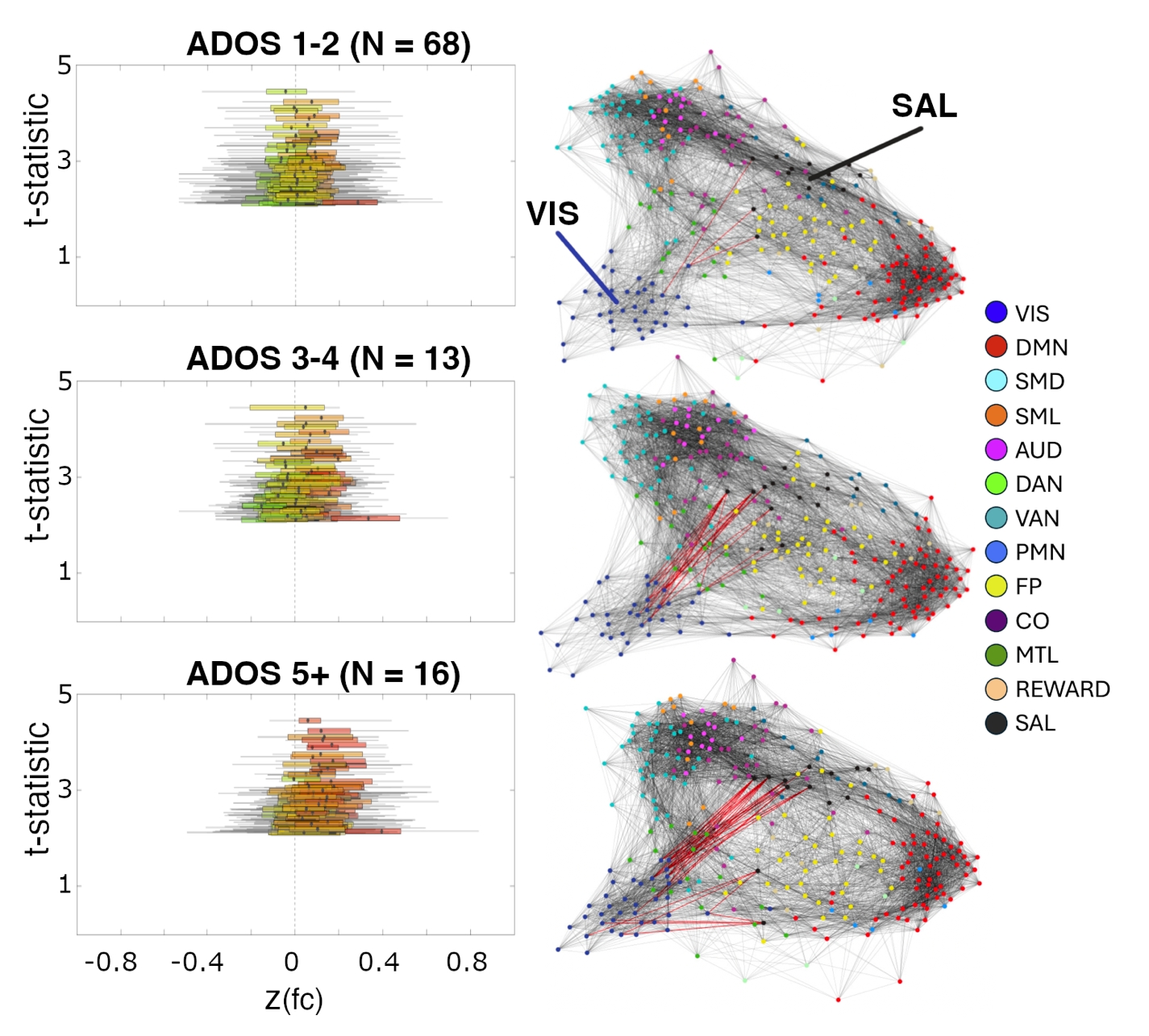


The functional connectivity and ROI-ROI network clusters were explored by stratifying subjects into low (1-2, N=68), medium (3-4, N=13), and moderate-to-high (5+, N=16) ADOS severity scores. Each box plot depicts the relationship between Fisher z-transformed functional connectivity, z(fc), and the t-statistic, for all positive SAL-VIS enrichment hits. The three groups show a positive shift in z(fc) as ADOS scores increase. The spring embedding of mean functional connectivity per group at a tie density of 42% show an increase in the number of SAL-VIS connections with increased ADOS score. The lack of connections for low ADOS scores agree with spring embedding results in healthy adults [5].

**Supplemental Figure 6: ORA Results for RRB, associations between RRB and SA findings**

There is a clear shift toward negative values **(a)** for *t-*statistics relating fc in SAL-VIS ROI-pairs (orange shaded area) to ADOS RRB scores when compared to all other ROI pairs and to the null *t* distribution (black line). **(b)** The correlation between SA and RRB *t*-statistics is *r =* -0.34, which is a consequence of the correlation between SA and RRB in the sample (*r =* 0.45). **(c)** We verified this correlation did not cause artifactual findings, by comparing results when analyzing SA and RRB jointly (*x-*axis) compared to SA alone (red) or RRB alone (orange). The *t*-statistics change slightly, as expected since the models are different, but the positive association between SA and SAL-VIS connectivity (red) and negative for RRB (orange) remain evident.

**Supplemental Figure 7: Framewise displacement and fc in SAL-VIS ROI pairs**


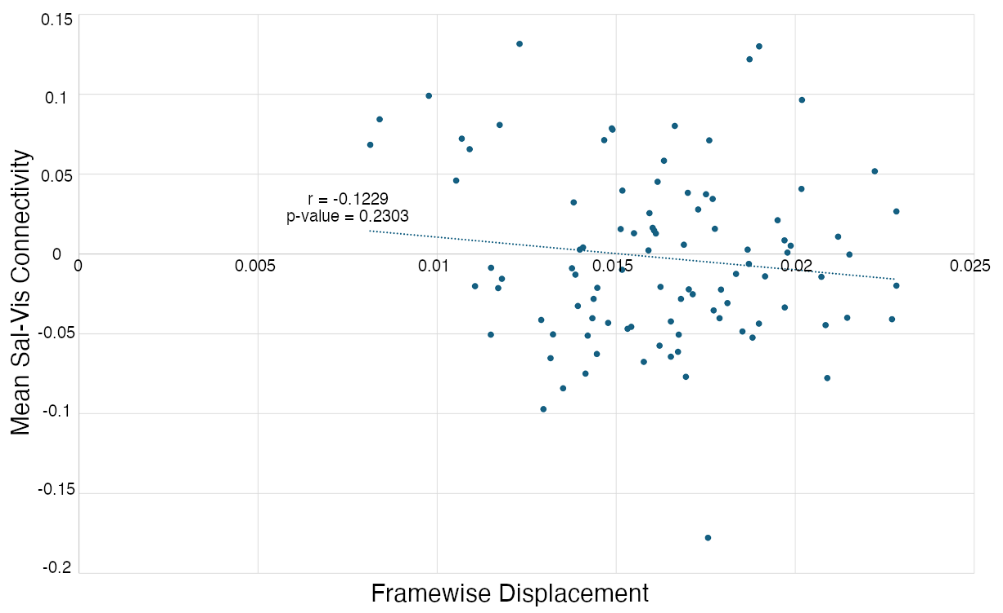


Per-subject mean censored framewise displacement plotted against the mean SAL-VIS functional connectivity. Movement dependency of the functional connectivity was not observed across the subjects (Pearson correlation = -0.1229, p-value = 0.2303).

**References**

1. Nordahl CW, Mello M, Shen AM, Shen MD, Vismara LA, Li D, et al. Methods for acquiring MRI data in children with autism spectrum disorder and intellectual impairment without the use of sedation. J Neurodev Disord. 2016;8:20.

2. Solari A, Finos L, Goeman JJ. Rotation-Based Multiple Testing in the Multivariate Linear Model. Biometrics. 2014;70:954–61.

3. Seitzman BA, Gratton C, Marek S, Raut RV, Dosenbach NUF, Schlaggar BL, et al. A set of functionally-defined brain regions with improved representation of the subcortex and cerebellum. Neuroimage. 2020;206:116290.

4. Diedrichsen J, Zotow E. Surface-Based Display of Volume-Averaged Cerebellar Imaging Data. PLoS ONE. 2015;10:e0133402.

5. Power JD, Cohen AL, Nelson SM, Wig GS, Barnes KA, Church JA, et al. Functional network organization of the human brain. Neuron. 2011;72:665–78.
